# Association of HbA1c With Cardiovascular Outcomes: The Role of Diabetes Duration and Cardiometabolic Factors

**DOI:** 10.7759/cureus.113829

**Published:** 2026-08-02

**Authors:** Aqsa Iqbal, Saanie Sulley, Chandra Kiran Jwala

**Affiliations:** 1 Internal Medicine, Southern Illinois University School of Medicine, Springfield, USA; 2 Health and Biomedical Informatics, National Healthy Start Association, Washington, DC, USA; 3 Internal Medicine, Southern Illinois Healthcare, Carbondale, USA

**Keywords:** biomarkers, diabetes mellitus, glycosylated hemoglobin (hba1c), national health and nutrition examination survey (nhanes), risk of cardiovascular diseases

## Abstract

Background

Glycated hemoglobin (HbA1c) is widely used as a marker of glycemic control and has been associated with cardiovascular disease (CVD); however, whether HbA1c is independently associated with prevalent CVD after accounting for traditional cardiometabolic risk factors remains uncertain.

Methodology

We conducted a cross-sectional analysis of 30,804 U.S. adults aged ≥18 years from the National Health and Nutrition Examination Survey (NHANES). Cardiovascular outcomes (coronary artery disease (CAD), myocardial infarction, and stroke) were defined using self-reported physician diagnoses. Associations between HbA1c and cardiovascular outcomes were evaluated using sequential logistic regression models with adjustment for demographic, metabolic, socioeconomic, and diabetes-related variables.

Results

Higher HbA1c levels were associated with increased odds of cardiovascular outcomes in unadjusted analyses. These associations were attenuated after adjustment for age and sex and were no longer statistically significant in fully adjusted models. Diabetes duration demonstrated a strong age-dependent gradient and contributed to attenuation of HbA1c effects. In stratified analyses, HbA1c was not significantly associated with outcomes in either individuals with diabetes or those without diabetes. Interaction analyses showed no consistent effect modification, except for a potential interaction between HbA1c and diabetes status for CAD.

Conclusions

The observed associations between HbA1c and prevalent cardiovascular outcomes were attenuated after adjustment for demographic, metabolic, socioeconomic, and diabetes-related variables. Because some adjustment variables may lie on the causal pathway, these findings should be interpreted cautiously and as observational associations rather than evidence of causality.

## Introduction

Cardiovascular disease (CVD) remains the leading cause of morbidity and mortality worldwide [1]. Diabetes mellitus is a major contributor to cardiovascular risk and is associated with accelerated atherosclerosis and increased incidence of coronary artery disease (CAD), myocardial infarction (MI), and stroke [2]. Glycated hemoglobin (HbA1c), which reflects average glycemic exposure over approximately two to three months, is widely used for the diagnosis and monitoring of diabetes [3]. Elevated HbA1c levels have been associated with increased cardiovascular risk in both diabetic and non-diabetic populations, supporting its role as a marker of cardiometabolic risk [4]. However, whether HbA1c is independently associated with prevalent cardiovascular outcomes after accounting for traditional cardiometabolic risk factors remains uncertain [5].

HbA1c is closely correlated with multiple determinants of cardiovascular risk, including obesity, lipid abnormalities, socioeconomic status, and diabetes duration, which may confound or mediate its association with CVD [6]. Recent studies highlight the importance of cumulative glycemic exposure and disease duration in driving cardiovascular risk [7]. Furthermore, the relationship between HbA1c and cardiovascular outcomes may vary across subgroups such as age, sex, and diabetes status, although findings remain inconsistent [8,9]. Therefore, comprehensive analyses incorporating sequential adjustment, diabetes duration, and subgroup evaluation are needed to better define the independent and context-specific role of HbA1c in cardiovascular risk assessment.

Although prior studies have demonstrated associations between HbA1c and CVD, uncertainty remains regarding whether these associations persist after sequential adjustment for demographic, metabolic, socioeconomic, and diabetes-related factors, including diabetes duration. In addition, relatively few studies have simultaneously examined stratified associations according to diabetes status and formal interaction analyses involving age, sex, and diabetes status within the same analytical framework. Therefore, we analyzed National Health and Nutrition Examination Survey (NHANES) data to address these knowledge gaps.

Aims and objectives

The primary objective was to evaluate whether HbA1c is independently associated with prevalent cardiovascular outcomes after sequential adjustment for demographic, metabolic, socioeconomic, and diabetes-related variables. Secondary objectives were to (1) evaluate the contribution of diabetes duration to the attenuation of HbA1c associations across sequential multivariable models; (2) determine whether the association between HbA1c and cardiovascular outcomes differs between individuals with and without diabetes; and (3) evaluate whether age, sex, or diabetes status modifies the association between HbA1c and cardiovascular outcomes using interaction analyses.

## Materials and methods

This cross-sectional study used publicly available NHANES data from 2014 to 2018. The study included U.S. adults aged ≥18 years with available HbA1c, cardiovascular outcome, and covariate data. Participants with missing key variables were excluded, resulting in a complete-case analytic sample of 30,804 individuals. A complete-case analysis was performed, and no imputation of missing data was undertaken.

HbA1c was analyzed as a continuous variable (per 1% increase). Diabetes status was defined based on self-reported physician diagnosis, and stratified analyses were performed comparing individuals with and without diabetes. The primary outcomes included CAD, MI, and stroke, defined based on self-reported physician diagnosis. A composite CVD outcome (any CVD) was defined as the presence of one or more of these conditions.

Covariates were selected a priori based on clinical relevance and included demographic and socioeconomic variables (age, sex, income-to-poverty ratio, and education level), metabolic factors (body mass index and high-density lipoprotein cholesterol), and diabetes-related variables (diabetes status and diabetes duration, calculated as the difference between age at interview and age at diagnosis).

Associations between HbA1c and cardiovascular outcomes were evaluated using logistic regression models, with results reported as odds ratios (ORs) and 95% confidence intervals (CIs). Sequential models included unadjusted, age- and sex-adjusted, and fully adjusted analyses. Stratified and interaction analyses were conducted to assess potential effect modification by age, sex, and diabetes status. Statistical significance was defined as a two-sided p-value <0.05.

Statistical analyses were performed using Python. Data management was performed using pandas, numerical computations using NumPy, logistic regression analyses using statsmodels, and figures were generated using Matplotlib and Seaborn. Because the merged analytic dataset available for this study did not retain the NHANES complex survey design variables (WTMEC2YR, SDMVSTRA, and SDMVPSU), survey-weighted analyses could not be performed. Accordingly, all analyses were conducted as standard unweighted logistic regression models.

## Results

Association of HbA1c with cardiovascular outcomes across sequential models

**Figure 1:**
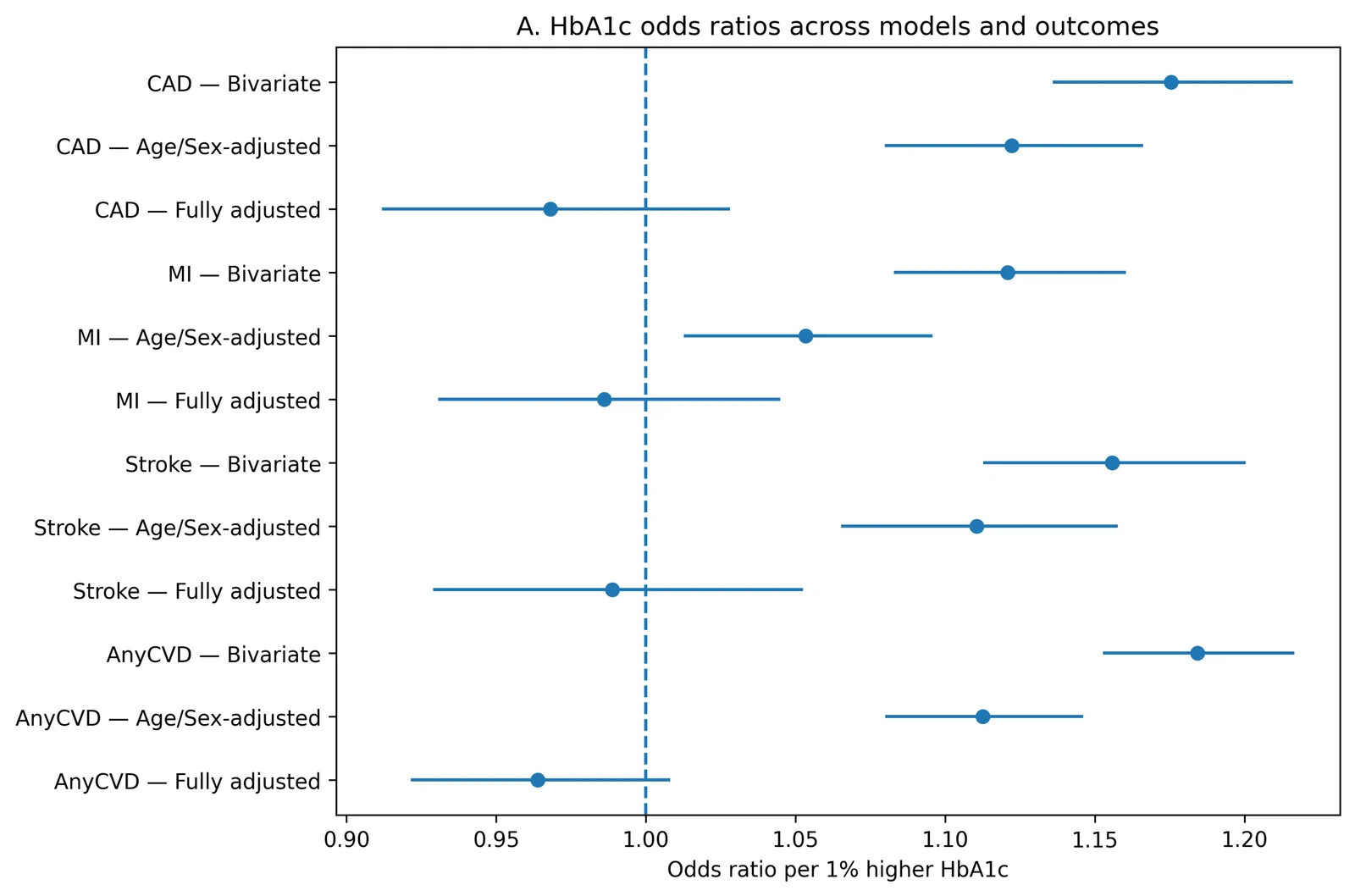
HbA1c associations across models and outcomes. Forest plot showing odds ratios (ORs) and 95% confidence intervals (CIs) for the association between glycated hemoglobin (HbA1c) (per 1% increase) and cardiovascular outcomes (coronary artery disease, myocardial infarction, stroke, and any cardiovascular disease) across sequential models (bivariate, age- and sex-adjusted, and fully adjusted). The dashed vertical line indicates the null value (OR = 1.0). Associations were attenuated and not statistically significant in fully adjusted models.

Higher HbA1c levels were associated with increased odds of cardiovascular outcomes in unadjusted analyses (Figure 1), where the corresponding ORs and 95% CIs are presented. Specifically, HbA1c was positively associated with CAD, MI, stroke, and composite CVD (any CVD), with all estimates reaching statistical significance (all 95% CIs excluding 1.0). This analysis was performed to evaluate the crude relationship between glycemic exposure and cardiovascular risk and to establish a baseline for assessing the impact of potential confounding factors.

After adjustment for age and sex, these associations remained statistically significant but were attenuated in magnitude, suggesting partial confounding by demographic factors (Figure 1). In fully adjusted models incorporating body mass index, high-density lipoprotein cholesterol, socioeconomic variables (income-to-poverty ratio and education), and diabetes-related factors (diabetes status and duration), HbA1c was no longer statistically significantly associated with CAD, MI, stroke, or any CVD, with all CIs crossing the null value (Figure 1).

Overall, these findings suggest that the crude associations between HbA1c and cardiovascular outcomes are substantially attenuated after accounting for covariates that may act as confounders and/or mediators, particularly diabetes-related factors. The attenuation observed across sequential models underscores the importance of considering underlying metabolic health, disease burden, and socioeconomic context when evaluating HbA1c as a cardiovascular risk marker. These results further suggest that HbA1c alone may have limited independent utility for cardiovascular risk association when comprehensive clinical and metabolic factors are taken into account.

Diabetes duration by age group

**Figure 2:**
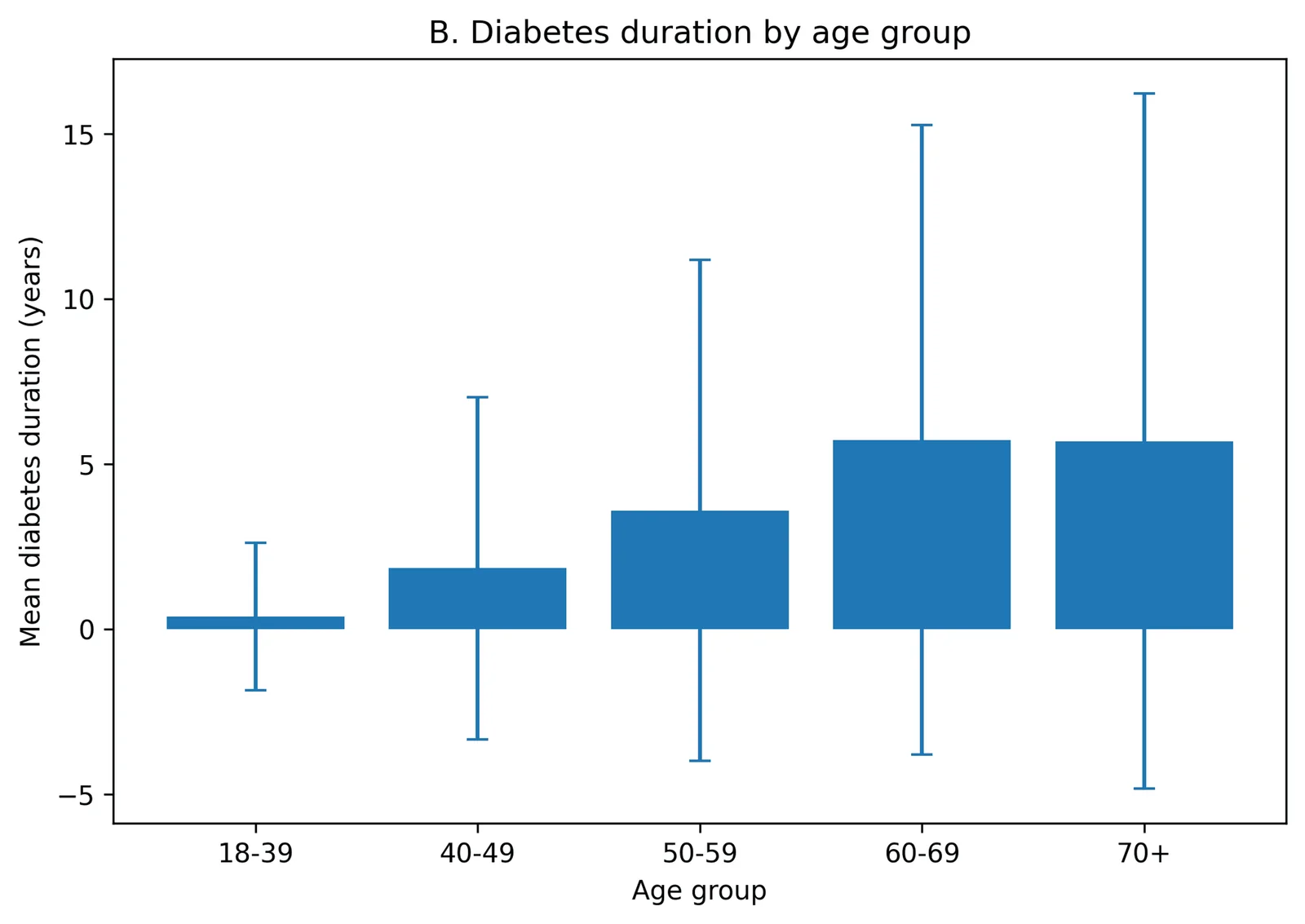
Diabetes duration by age group. Bar plot showing mean diabetes duration (years) across age groups, with error bars representing variability within each group. Diabetes duration increases progressively with age, with greater dispersion observed in older age groups.

Figure 2 illustrates mean diabetes duration across age groups. This analysis was conducted to characterize the distribution of diabetes duration across the life course and to provide context for its inclusion as a key covariate in multivariable models evaluating the association between HbA1c and cardiovascular outcomes. A progressive increase in mean diabetes duration was observed with advancing age.

Participants aged 18-39 years had the shortest mean duration, approximating near-zero years (Figure 2). Mean duration increased in a graded manner among individuals aged 40-49 and 50-59 years, reaching approximately two to four years. Older age groups exhibited longer diabetes duration, with mean values of approximately five to six years among participants aged 60-69 and ≥70 years. Error bars indicate substantial variability within each age group, with wider dispersion in older populations, suggesting greater heterogeneity in diabetes duration (Figure 2).

Overall, these findings indicate that older individuals tend to have longer diabetes duration. The strong age-duration relationship supports including duration as a key covariate, reflecting cumulative disease burden that may influence cardiovascular risk and contribute to attenuation of HbA1c associations in adjusted models.

HbA1c associations stratified by diabetes status

**Figure 3:**
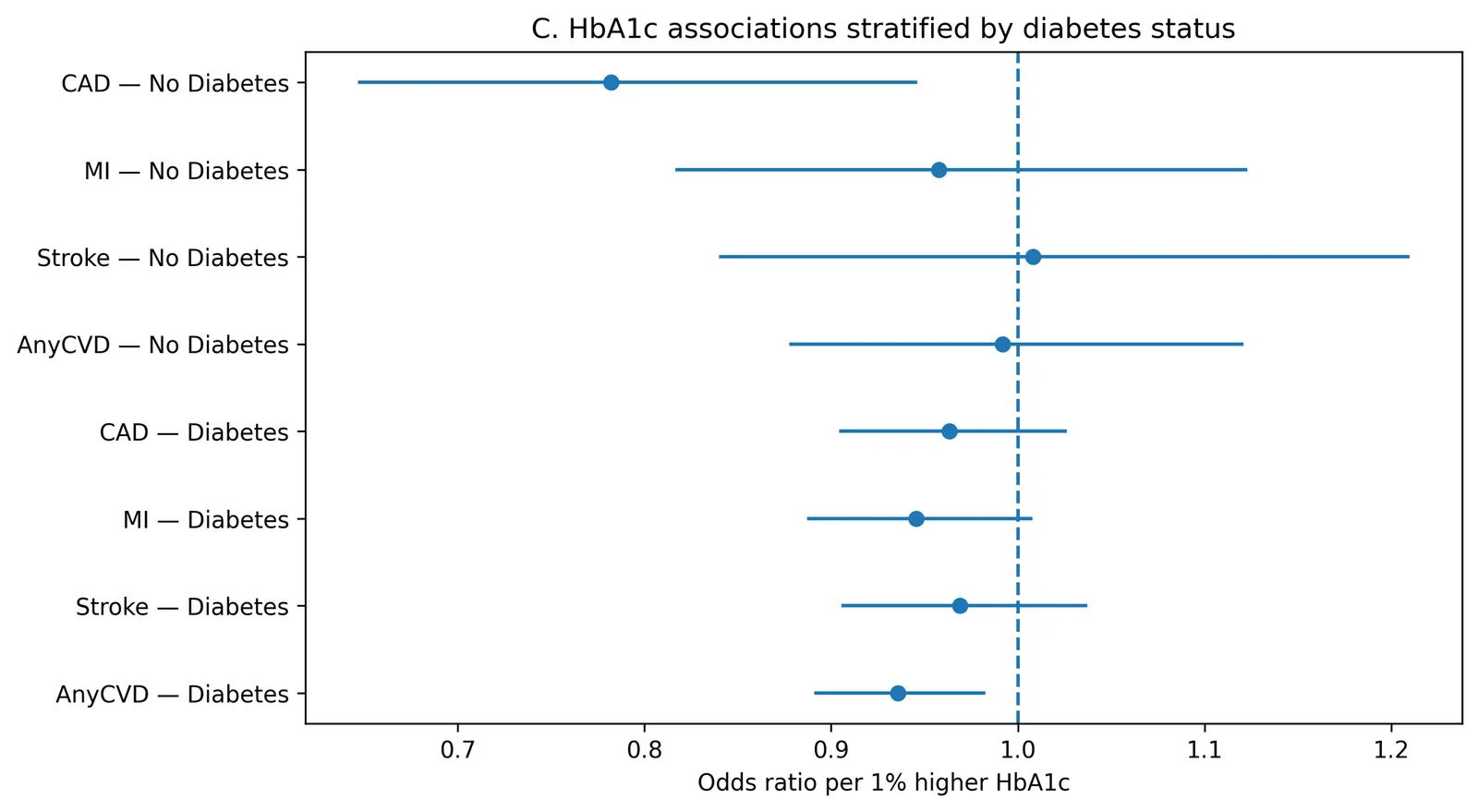
HbA1c associations stratified by diabetes status. Forest plot showing odds ratios (ORs) and 95% confidence intervals (CIs) for the association between glycated hemoglobin (HbA1c) (per 1% increase) and cardiovascular outcomes (coronary artery disease (CAD), myocardial infarction (MI), stroke, and any cardiovascular disease (CVD)) in individuals with and without diabetes. The dashed vertical line indicates the null value (OR = 1.0). No statistically significant associations were observed in either group.

Figure 3 presents the association between HbA1c (per 1% increase) and cardiovascular outcomes stratified by diabetes status in fully adjusted models. This stratified analysis was performed to evaluate whether the relationship between HbA1c and cardiovascular outcomes differs by diabetes status, given differences in glycemic range, disease pathophysiology, and cumulative metabolic exposure between individuals with and without diabetes.

Among individuals with and without diabetes, HbA1c was not significantly associated with cardiovascular outcomes after multivariable adjustment (Figure 3). ORs for CAD, MI, stroke, and composite CVD (any CVD) were close to the null value, with all CIs crossing 1.0. While the estimate for CAD in individuals without diabetes was below 1.0, it was imprecise and not statistically significant.

These findings suggest that, after accounting for metabolic, socioeconomic, and diabetes-related factors, HbA1c is not significantly associated with cardiovascular outcomes within either diabetes stratum. The lack of statistically significant association in individuals with diabetes should be interpreted cautiously because adjustment for diabetes-related variables, including diabetes duration, may have contributed to attenuation of the observed associations. In individuals without diabetes, the relatively narrow distribution of HbA1c may limit its ability to discriminate cardiovascular risk.

Overall, these results highlight that the relationship between HbA1c and cardiovascular outcomes is context-dependent and may be largely explained by underlying disease burden and associated cardiometabolic factors rather than HbA1c alone (Figure 3).

Interaction effects between HbA1c and demographic factors

**Figure 4:**
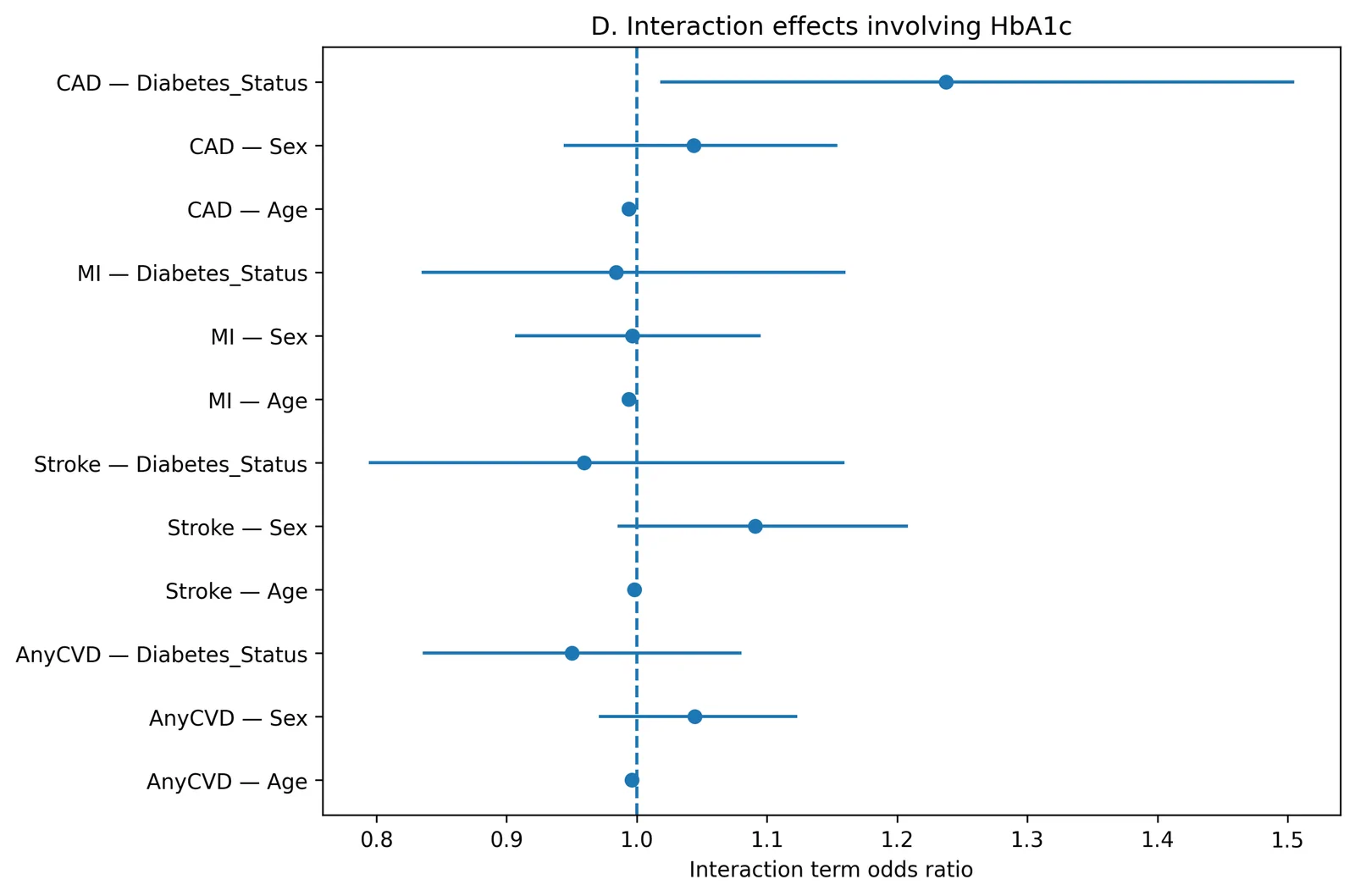
Interaction effects between HbA1c and demographic factors on cardiovascular outcomes. Forest plot showing interaction term odds ratios (ORs) and 95% confidence intervals (CIs) for the association between glycated hemoglobin (HbA1c) and cardiovascular outcomes (coronary artery disease (CAD), myocardial infarction (MI), stroke, and any cardiovascular disease (CVD)) across subgroups defined by age, sex, and diabetes status. The dashed vertical line indicates the null value (OR = 1.0). No significant effect modification was observed for age or sex, while a potential interaction with diabetes status was noted for CAD.

Figure 4 presents interaction analyses evaluating whether the association between HbA1c (per 1% increase) and cardiovascular outcomes differs by age, sex, and diabetes status. Interaction term ORs with corresponding CIs are shown, with the dashed vertical line representing the null value (OR = 1.0) (Figure 4).

No statistically significant interactions were observed between HbA1c and age, as CIs for all outcomes crossed the null value. A statistically significant interaction between HbA1c and diabetes status was observed for CAD, indicating that the association between HbA1c and CAD differs between individuals with and without diabetes (Figure 4). No significant interactions with diabetes status were observed for MI, stroke, or composite CVD (any CVD). No statistically significant interactions were observed between HbA1c and sex, as all confidence intervals crossed the null value (Figure 4).

Overall, these findings suggest limited evidence of effect modification in the association between HbA1c and cardiovascular outcomes, except for a potential interaction with diabetes status for CAD.

## Discussion

In this study, higher HbA1c levels were associated with increased odds of cardiovascular outcomes in unadjusted analyses, but these associations were attenuated and no longer statistically significant after multivariable adjustment. This finding suggests that the crude relationship between HbA1c and CVD is largely explained by confounding and/or mediation by metabolic, socioeconomic, and diabetes-related factors. Similar attenuation has been reported in contemporary cohort studies, in which adjustment for cardiometabolic risk factors substantially reduces the independent association value of HbA1c [10].

Recent large-scale analyses have also questioned the independent role of HbA1c in cardiovascular risk prediction. For example, a population-based study by Rawshani et al. demonstrated that while HbA1c is associated with cardiovascular events in unadjusted models, its predictive value diminishes after accounting for traditional risk factors and comorbidities [11]. Likewise, analyses from the ADVANCE trial showed that inclusion of metabolic variables and comorbidity burden attenuated HbA1c associations with incident CVD, reinforcing the importance of comprehensive risk modeling [12].

Our findings should be interpreted in the context of the broader prospective literature demonstrating associations between long-term glycemic control and cardiovascular risk. Differences in study design, outcome definitions, duration of follow-up, and covariate adjustment strategies may account for differences between the present cross-sectional analysis and prior longitudinal studies. Accordingly, our findings should be viewed as complementary to, rather than replacing, evidence derived from prospective investigations.

An important feature of the present study was the evaluation of diabetes duration as a clinically relevant covariate within the sequential regression models. This finding is consistent with prior evidence suggesting that cumulative glycemic exposure increases with advancing age and may contribute to cardiovascular risk. Previous longitudinal studies have reported that longer diabetes duration is associated with higher risk of cardiovascular events compared with single-point HbA1c measurements, highlighting the importance of chronic exposure over time [13].

The attenuation of HbA1c associations after adjusting for diabetes duration in our study may reflect partial mediation, as duration lies on the causal pathway linking hyperglycemia to vascular damage. Mechanistic studies have shown that prolonged exposure to hyperglycemia promotes endothelial dysfunction, oxidative stress, and inflammation, which contribute to atherosclerosis progression [14]. These findings support the concept that HbA1c, when measured at a single time point, may not adequately capture cumulative vascular injury.

Stratified analyses in our study further demonstrated that HbA1c was not significantly associated with cardiovascular outcomes in either diabetic or non-diabetic individuals after adjustment. This is consistent with prior evidence demonstrating that in individuals with diabetes, cardiovascular risk is more strongly influenced by disease duration and cumulative metabolic burden than by single-point glycemic measures [15].

Interaction analyses revealed no significant effect modification by age or sex and only limited interaction by diabetes status for CAD, indicating that the lack of independent association is broadly consistent across subgroups. This aligns with subgroup analyses from large-scale cohort studies and individual-participant meta-analyses demonstrating minimal heterogeneity in HbA1c-CVD associations across age and sex strata, with attenuation of effect estimates after adjustment for confounding variables [2,4].

From a clinical perspective, these findings suggest that HbA1c alone may have limited utility as an independent predictor of cardiovascular risk when comprehensive cardiometabolic factors are considered. Contemporary guidelines increasingly emphasize multifactorial risk assessment, incorporating clinical, metabolic, and demographic variables rather than relying on glycemic markers alone [3].

The present findings have important clinical implications. Although HbA1c is widely used in clinical practice, our results suggest that it may have limited utility as an independent association of cardiovascular risk when considered in isolation. Instead, cardiovascular risk assessment should incorporate a comprehensive evaluation of metabolic, demographic, and disease-related factors, including diabetes duration, which may better reflect cumulative glycemic exposure. These findings support current guideline recommendations emphasizing multifactorial risk assessment and highlight the need to interpret HbA1c within the broader context of cardiometabolic health rather than as a standalone marker of cardiovascular risk. Future research should focus on longitudinal measures of glycemic exposure, including time-updated HbA1c and glycemic variability, as well as integrated risk models that better capture cumulative metabolic risk.

Strengths

Strengths of this study include the large NHANES sample, evaluation of multiple cardiovascular outcomes, comprehensive sequential multivariable adjustment incorporating demographic, metabolic, socioeconomic, and diabetes-related variables, formal interaction analyses, and stratified analyses according to diabetes status, age, and sex. Together, these approaches provide a comprehensive assessment of the association between HbA1c and cardiovascular outcomes across clinically relevant subgroups.

Limitations

This study has several limitations. First, the cross-sectional design precludes determination of temporal or causal relationships, and reverse causation cannot be excluded because HbA1c and cardiovascular outcomes were assessed at the same time. Second, cardiovascular outcomes were based on self-reported physician diagnoses and therefore may be subject to recall or misclassification bias, which would likely attenuate observed associations. Third, residual confounding from unmeasured variables, including medication use (such as glucose-lowering therapies, statins, and antihypertensive agents), smoking, renal function, physical activity, inflammatory markers, and family history of CVD, cannot be excluded despite multivariable adjustment. Fourth, diabetes duration was included in the fully adjusted models and may represent overadjustment if it lies on the causal pathway between chronic hyperglycemia and CVD, potentially attenuating true associations. Fifth, because the merged analytic dataset did not retain the NHANES complex survey design variables (WTMEC2YR, SDMVSTRA, and SDMVPSU), analyses were conducted without survey weighting and therefore should not be interpreted as nationally representative estimates. Finally, the findings are limited to the non-institutionalized U.S. civilian population represented by NHANES and may not be generalizable to other populations.

## Conclusions

These findings demonstrate that the observed association between HbA1c and cardiovascular outcomes was substantially attenuated after adjustment for demographic, metabolic, socioeconomic, and diabetes-related variables. Because of the cross-sectional design and the inclusion of variables that may lie on the causal pathway, these findings should be interpreted as observational associations rather than evidence of causality. Additional prospective studies are needed to clarify the temporal relationship between HbA1c and CVD.
